# Metacognitive Therapy for Individuals at High Risk of Developing Psychosis: A Pilot Study

**DOI:** 10.3389/fpsyg.2019.02741

**Published:** 2020-01-17

**Authors:** Sophie Kate Parker, Lee D. Mulligan, Philip Milner, Samantha Bowe, Jasper E. Palmier-Claus

**Affiliations:** ^1^Psychosis Research Unit, Greater Manchester Mental Health NHS Foundation Trust, Manchester, United Kingdom; ^2^Youth Mental Health Research Unit, Greater Manchester Mental Health NHS Foundation Trust, Manchester, United Kingdom; ^3^Division of Psychology and Mental Health, University of Manchester, Manchester, United Kingdom; ^4^Spectrum Centre for Mental Health Research, Lancaster University, Lancaster, United Kingdom; ^5^Lancashire & South Cumbria NHS Foundation Trust, Lancashire, United Kingdom

**Keywords:** metacognition, metacognitive therapy, at risk mental states, psychosis, cognitive attentional syndrome

## Abstract

Developing effective interventions for preventing first episode psychosis have been an important research focus in the last decade. Cognitive behavioral therapy is a currently indicated treatment for people at ultra-high risk of psychosis, however, access and resource issues limit its delivery within the NHS. Treatments which partial out potential active ingredients and are aimed at a range of psychological difficulties seen within this population have the potential to be more efficacious and efficient. We conducted a single-arm exploratory pilot trial, designed to investigate the feasibility and acceptability of Metacognitive therapy for individuals at ultra-high risk (UHR) of developing psychosis. Trial uptake was good, with 11 out of 12 referred individuals meeting for an eligibility assessment (one individual was excluded prior to the assessment). Of these, 10 individuals were eligible and included in the trial. Retention to treatment was high with 80% treatment adherence gained and an overall average of 8 sessions completed. All participants were offered follow-up assessments immediately post-treatment and at 6 months, which comprised measures of psychotic like experiences, anxiety and depression, and metacognitive processes implicated in the model. Retention to the post-treatment (12-week) follow-up was good, with 80% completion; however retention to the 6-month follow-up was lower at 60%. Clinically significant results were observed in psychotic like experiences, anxiety, depression and functioning with medium to large effect sizes. Measures related to beliefs and processes targeted within MCT showed clinically significant change with medium to large effect sizes. Our results suggest that MCT based upon a specific metacognitive model for individuals meeting ARMS criteria may be an important treatment target and warrants further attention. Limitations and possible focuses for future research are discussed.

**Registration:** ISRCTN53190465 http://www.isrctn.com/ISRCTN53190465.

## Introduction

Given the cost to individuals, families and services of psychosis, it is unsurprising that there has been great emphasis in research on the prevention of the development of a first episode. There are reliable and valid criteria available to identify help-seeking individuals in diverse settings who are at high risk of developing psychosis. Yung et al. (1998) developed operational criteria to identify three subgroups possessing an “at risk mental state” (ARMS) for psychosis. Two subgroups specify state risk factors, defined by the presence of either transient psychotic symptoms, called Brief Limited Intermittent Psychotic Symptoms (BLIPS) or attenuated (subclinical) psychotic symptoms (AS). The other subgroup comprises trait-plus-state risk factors, operationally defined by the presence of diminished functioning plus a first-degree relative with a history of psychosis. All subgroups are within a specified age range known to be at greatest risk for the onset of psychosis, and all participants in studies of ARMS to date have been help-seeking.

In addition to identification, developing effective interventions to prevent or delay transition to psychosis have been an important research focus, given the potential benefits for symptoms, recovery and other outcomes. To date, there have been eight randomized controlled trials, each using similar operational definitions of ARMS, that have investigated antipsychotic medication, omega-3 polyunsaturated fatty acids and/or psychological interventions. The studies were conducted in Australia (McGorry et al., 2002; Yung et al., 2011), North America (McGlashan et al., 2006; Addington et al., 2011), the United Kingdom (Morrison et al., 2004a, 2006, 2012), the Netherlands (van der Gaag et al., 2012; Ising et al., 2016) and Austria (Amminger et al., 2010). Significant benefits at 12 months post-intervention were found for both cognitive behavioral therapy (Morrison et al., 2004a, 2006; van der Gaag et al., 2012) and omega-3 polyunsaturated fatty acids (Amminger et al., 2010). Therefore, at present, the recommended psychological treatment for young people at high risk of developing psychosis is cognitive behavioral therapy. The treatment duration indicated by the current evidence base (see Stafford et al., 2013) may be up to 26 sessions if following an appropriate manual for cognitive behavioral therapy for young people at risk of psychosis (e.g., French and Morrison, 2004). However, only a small number of young people meeting the at-risk of psychosis criteria are offered such indicated interventions in the NHS. In 2016 an audit found that only 41% of clients under EIS nationwide are offered CBTp within 6 months of acceptance into EIT (Health Quality Improvement Partnership and the Royal College of Psychiatrists, 2016). The audit operationalized an offer of CBTp as an offer of 16 sessions, delivered by appropriately trained and supervised therapists.

The United Kingdom access and waiting time standards (Nhs England, the National Collaborating Centre for Mental Health, and the National Institute for Health, and Care Excellence, 2016), which came into force from 1st April 2016, ensures that Early Interventions Services offer assessments for ARMS. However, the ability for NHS services to offer indicated interventions in line with the research protocols for people meeting ARMS criteria is limited given their stretched resources. If we can find out more about the active ingredients in psychological interventions, we may be able to make our interventions more efficacious and efficient. There has also been a call for more intervention trials aimed at the range of psychopathology observed in those at high risk of developing psychosis, to inform best care practices (Carpenter, 2018).

The cognitive model developed by Morrison (2001) implicates both cognitive and metacognitive processes in the development of psychosis. It is possible that using a specifically metacognitive approach could be a more efficient, quicker treatment compared with a mixed model. Studies have demonstrated a role of metacognitive beliefs and processes in the development and maintenance of psychosis (Smári et al., 1994; Morrison et al., 2002, 2004b, 2005, 2007; Morrison and Wells, 2003, 2007; Sellers et al., 2018). Metacognitive processes are also prevalent and important in those meeting criteria for ARMS (e.g., Morrison et al., 2007; Brett et al., 2009; Debbané et al., 2009; Barkus et al., 2010; Debbané et al., 2012; Palmier-Claus et al., 2013; Cotter et al., 2017).

Different metacognitive models and the approaches they derive for the treatment of psychosis have been summarized by Lysaker et al. (2018). These models are distinct and underpinned by different theoretical perspectives. The approach described here is underpinned by the S-REF (self-regulatory executive function) model proposed by Wells and Matthews (1994, 1996) and is not to be confused with metacognition as defined within other models being used within the area of psychosis (e.g., Lysaker et al., 2005). Wells and Matthews propose that it is not the occurrence of mental events (i.e., negative thoughts and emotion) that give rise to prolonged distress, but the resulting perseverative thinking style called the cognitive attentional syndrome (CAS). The CAS is comprised of strategies aimed at managing distressing thoughts and emotions which include worry, rumination, threat monitoring thought control strategies and maladaptive coping behaviors such as avoidance and reassurance seeking (Wells, 2008). The model implicates a central role of the CAS which becomes employed in response to negative thoughts and feelings causing an extension to psychological distress and worsening and extending negative affect. The S-REF model hypothesizes that CAS activity is promoted by underlying metacognitive beliefs both positive and negative in orientation. For example, people hold positive beliefs such as “*worrying will help me to be prepared*” and on the other hand negative beliefs about the uncontrollability and danger of thoughts and feelings such as “I *cannot control my worrying once it begins.*”

A large body of evidence implicates a central role of metacognition in numerous mental health problems including generalized anxiety disorder, social anxiety, depression, PTSD and psychosis (Wells, 1995; Clark and Wells, 1995; Morrison, 2001; Papageorgiou and Wells, 2001), and metacognitive therapies for such problems are being applied successfully (Wells, 2000, 2008; Normann and Morina, 2018). A metacognitive model of the positive symptoms of psychosis has been developed (Morrison, 2001). This evidence-based model predicts that metacognitive therapy may help to reduce psychotic like symptoms and target symptoms of co-morbid emotional disorders.

Where metacognitive processes are implicated, it is likely that specific metacognitive therapy for people at high risk of developing psychosis will be effective. Recent evidence has suggested that metacognitive therapy (MCT; Wells and Matthews, 1994, 1996) is a useful alternative to CBT for understanding and treating disorders such as generalized anxiety disorder, post-traumatic stress disorder, obsessive compulsive disorder and depression (Wells and King, 2006; Wells, 2008; Normann and Morina, 2018). MCT is a shorter treatment than traditional CBT, requiring around 6–8 sessions for symptom improvement (Wells, 2008). It has low drop-out rates and appears to be well-tolerated in emotional disorders. MCT could be particularly useful as an alternative treatment for young people at high risk of developing psychosis as it does not directly challenge the patients’ belief systems, but rather focuses on the process, of thinking. It is also potentially generalizable to the other axis I disorders which have high co-morbidity within young at-risk individuals (e.g., Leicester et al., 2002).

A single arm feasibility study of 12 sessions of MCT for individuals with psychosis has been conducted (Morrison et al., 2014). This study successfully recruited 10 participants and adherence to MCT was shown to be acceptable; all participants received at least one session and 9/10 received 6 sessions or more (a mean of 10.6). The treatment demonstrated encouraging within-subjects effect sizes on positive symptoms (Cohen’s *d* = 1.27) and delusional beliefs (Cohen’s *d* = 0.71), and on negative symptoms (Cohen’s *d* = 0.62), for which the evidence base in support of CBT is sparse. These positive results for individuals who by definition have a more ‘serious’ symptom profile suggests that MCT may also be useful for people at high risk of developing psychosis.

This pilot trial provides a preliminary investigation into the acceptability and feasibility of MCT for people meeting ARMS criteria who were experiencing distressing symptoms. It also provides an initial investigation into the efficacy of MCT in producing relief from psychotic like symptoms. In line with standard feasibility aims, the objectives of this study were to assess recruitment rate and to examine the appropriateness, feasibility and acceptability of the intervention and measures. It was hypothesized that MCT would produce symptom relief from unusual or overvalued beliefs (e.g., paranoia) and perceptual experiences (e.g., hallucinations), defined by significantly reduced CAARMS scores at both end of treatment and follow-up.

## Materials and Methods

### Design

This study was a single-arm exploratory trial, designed to investigate the feasibility and acceptability of MCT for individuals at ultra-high risk (UHR) of developing psychosis. A National Research Ethics Committee approved the study prior to commencing data collection (13/NW/0238).

### Participants

All participants were being seen by NHS services specifically developed to work with people at high risk of developing psychosis [e.g., an Early Detection and Intervention Team (EDIT) or Early Intervention Service (EIS)] in the community. All participants met criteria for being at UHR of developing psychosis as operationally defined by the Comprehensive Assessment of At-Risk Mental States (CAARMS: Yung et al., 2005). NHS patients are typically referred to such services by primary care clinicians e.g., General Practitioners (GPs) or primary care psychology services, and should be offered assessment, ongoing monitoring of their mental health and Cognitive Behavioral Therapy (CBT).

Our exclusion criteria were: (i) a moderate to severe learning disability; (ii) a neurological impairment of organic origin (e.g., head injury or dementia); (iii) limited command of the English language, sufficient to impede the use of standardized assessments or accessibility of therapy; (iv) currently receiving inpatient care; (v) judged by their case manager to be clinically unstable over the 4 weeks prior to participation; (vi) taking prescribed antipsychotic medication; or (vii) a primary diagnosis of substance dependency.

### Measurements

#### Primary Outcomes

The primary outcome measure was to assess feasibility and acceptability outcomes including levels of recruitment into the trial, retention of participants across baseline assessment, intervention and follow-up periods, number of “drop outs,” defined as an individual who attended three or less therapy sessions, and adherence to the therapy protocol.

#### Secondary Outcomes

A number of secondary outcomes were included. The CAARMS (Yung et al., 2005); a semi-structured interview credited as the gold-standard for assessing “at risk” symptoms. The CAARMS interview comprises six subscales assessing unusual thought content, non-bizarre ideas, perceptual abnormalities, disorganized speech, aggressive behavior, and suicidality over the previous month. Each subscale is rated on two seven-point scales according to the severity and frequency of any endorsed symptom. Scores for each subscale are derived from the product of the severity (0–6) and frequency scores (0–6). A number of studies have shown that the CAARMS has excellent inter-rater reliability, in addition to concurrent, discriminant and predictive validity (Yung et al., 2005).

The Global Assessment of Functioning (GAF; Hall, 1995) was used to measure personal, social and psychological functioning. The GAF is a semi-structured interview measure used in conjunction with the CAARMS and scores range from 0 to 100, with higher scores indicating greater global functioning. The GAF has been widely validated (Jones et al., 1995) and has been used extensively in studies examining UHR samples (Hartmann et al., 2016).

Anxiety and depression symptoms were measured using the Hospital Anxiety and Depression Scale (HADS; Zigmond and Snaith, 1983), a 14 item self-report questionnaire providing separate total scores for anxiety and depression severity. All items are rated on four-point scales in reference to the previous week and higher scores indicate greater symptom severity. The HADS is considered to possess adequate to good properties of sensitivity, case-finding, concurrent validity and internal consistency (Bjelland et al., 2002). It is a brief and straightforward self-report measure which assesses both depression and anxiety (Hansson et al., 2009) thus reducing the burden of completion for participants. It has been used in similar populations to ours, such as adolescents, young people and adults with psychosis (Bernard et al., 2006; White et al., 2011; Pyle et al., 2019), therefore allowing comparability of our results with similar studies.

Metacognitive beliefs were assessed using the metacognitive questionnaire (MCQ-30; Wells and Cartwright-Hatton, 2004). This is a 30-item measure comprising five subscales: positive beliefs about worry; negative beliefs about uncontrollability and danger of extended processing; cognitive confidence; cognitive self-consciousness; and need to control thoughts. All items are rated on four-point Likert scales ranging from 1 (“do not agree”) to 4 (“agree very much”). The MCQ has strong psychometric properties and has been used in studies examining UHR populations (Morrison et al., 2007; Bright et al., 2018).

Activation of the CAS was measured using the CAS scale (CAS-1; Wells, 2008); a 16-item questionnaire assessing worry, threat monitoring, strategies in response to negative thoughts or feelings, and metacognitive beliefs about extended processing and thought control strategies. For this study, only items assessing degrees of worry and threat monitoring were included. Both items are rated on eight-point scales ranging from 0 (“none of the time”) to 8 (“all of the time”), in reference to experiences during the previous week. The CAS-1 has strong psychometric properties, including good internal consistency, concurrent and predictive validity (Sellers et al., 2018).

Appraisals of voice hearing were measured using the Interpretations of Voices Inventory (IVI; Morrison et al., 2002), a 26-item self-report questionnaire assessing positive and negative hypothetical interpretations of voices. All items are rated on four-point Likert scales ranging from 1 (“not at all”) to 4 (“very much”). The IVI comprises three subscales: metaphysical beliefs, positive beliefs and beliefs about loss of control. The IVI is reliable and valid for use with people defined as high in psychosis-proneness (Morrison et al., 2004b).

Metacognitive beliefs about paranoia were assessed using the Beliefs about Paranoia Scale – Short form (BAPS; Gumley et al., 2011), an 18-item self-report questionnaire measuring conviction in positive and negative interpretations. Each item is measured on a four-point Likert scale ranging from 1 (“not at all”) to 4 (“very much”). The BAPS can be subdivided into three scales: negative beliefs about paranoia, positive beliefs about paranoia as a survival strategy and normalizing beliefs. The BAPS has strong psychometric properties (Morrison et al., 2011) and has been used with UHR samples (Morrison et al., 2015).

### Procedure

All participants were recruited from EDIT and EIS teams. Individuals were identified and approached by members of their clinical teams to participate in this study. Participants completed a battery of assessments (CAARMS, HADS, MCQ-30, CAS-1, IVI, BAPS) at baseline (pre-therapy), end of therapy (3 months) and 6 months post-therapy. Two clinical measures (HADS, CAS-1) were administered prior to each therapy session. All assessments were conducted by a trainee clinical psychologist or qualified clinical psychologist with extensive training and experience of administering the CAARMS. Throughout the study, all CAARMS scores were reviewed in supervision and ratings were finalized through group discussion. To reduce bias, all follow-up assessments (end of therapy and 6-months post therapy) were conducted by a therapist who was not involved in the delivery of therapy, for each respective participant.

### Intervention

The MCT intervention consisted of 12 sessions over a period of 12 weeks following baseline assessment, and followed the treatment manual developed by Wells (2009). We adapted the metacognitive model of generalized anxiety disorder (Wells, 1995) for use with UHR individuals, in a similar way to the therapy previously described for people with a diagnosis of schizophrenia (Hutton et al., 2014; Morrison et al., 2014). The metacognitive model asserts that psychological distress results from extended processing in response to negative cognitions (comprising thoughts, images, voices etc.). Examples of extended processing include worry, rumination and unhelpful thought control strategies, collectively termed as the CAS (Wells and Matthews, 1996). MCT aims to reduce the CAS by exploring new ways of responding to worrying thoughts and modifying metacognitive beliefs, which contribute to worry, rumination and distress. The MCT intervention consisted of:

1. Case conceptualization via assessment of a recent episode of worry in which the person became distressed by the worry. This allows for the generation of an idiosyncratic version of the metacognitive model depicting positive and negative metacognitive beliefs as well as coping behaviors and thought control strategies.
2. Socialization to the metacognitive model through sharing the conceptualization and exploring the role of beliefs about worry via verbal and behavioral experiment strategies as well as the effects of behaviors.
3. Questioning and challenging beliefs about uncontrollability via verbal techniques and loss of control experiments including the introduction of detached mindfulness and worry postponement.
4. Challenging beliefs about the danger of worry via verbal reattribution and consolidating learning via behavioral experiments.
5. Challenging positive metacognitive beliefs about worry and generating alternative ways of responding to internal events.
6. Developing and reinforcing new plans for processing worry where old plans and new plans are described side by side.
7. Relapse prevention.

### Therapists

Four therapists delivered the intervention. All therapists received training in the MCT manual and received weekly supervision to ensure adherence to the model. With participant consent, supervisors reviewed audio recordings of therapy to maximize fidelity.

### Statistical Analysis

Data were analyzed using Stata 14.0 (Stata Corporation, 2011). Emphasis was placed on descriptive and summary statistics, and flow across the different stages of the trial. In the absence of normally distributed data, Wilcoxon signed-rank tests assessed differences between assessment scores at baseline and post-therapy, and at baseline and 6-month follow-up. Summary effect sizes were also calculated (Cohen’s *D*) using SD pre as the pre-test value provides an arguably better estimate of the true population value for within-subjects designs. This value is also thought to provide a better comparison to the d statistic in paired-design experiments thereby making it useful in meta-analysis (Cummings, 2012). Regression, with clustering at the participant level (Rogers, 1993), was used to explore the relationship between meta-cognitive beliefs and worry (CAS-1) across the sessional measures in a ‘long-form’ of the data.

## Results

**FIGURE 1:**
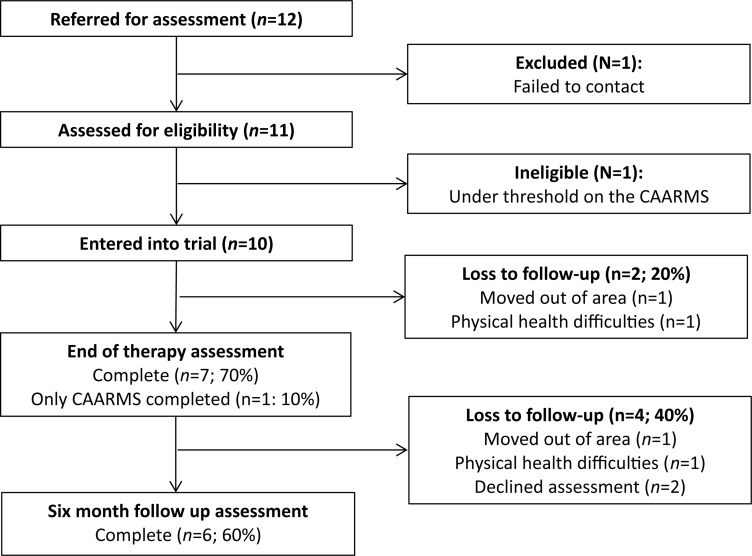
CONSORT diagram for flow at the different stages of the trial.

The trial ended in December 2015 with a final sample size of 10. Demographic information for the sample is presented in Table 1. The consort diagram (Figure 1) shows the size of the sample across the different stages of the study. Uptake of the trial was good. Out of 12 referred individuals, 11 met for baseline eligibility assessments. One individual was excluded prior to assessment, and one was found to be ineligible post-baseline assessment due to not meeting CAARMS threshold criteria. Adherence to MCT was adequate with participants completing an average of 8.0 sessions (SD, 4.4; range, 1–12.). Two participants only completed one session due to changes in employment and unstable life circumstances, respectively. Eighty percent of participants completed the post-treatment (12-week) assessment, (one of these participants only completed the CAARMS assessment and not the questionnaire measures) whereas 60% repeated these assessments at the 6-month follow-up. This was due to clients moving out of area (*n* = 1), declining assessment (*n* = 2), and physical health complications (*n* = 1).

**TABLE 1:** Demographic information at baseline.

Age, mean *SD*	22.8 (4.0)
Male: Female, *n*	6:4
Ethnicity	
White British, *n*	8
Asian Indian, *n*	1
Asian other, *n*	1
In employment and training: NEET ratio, *n*	1:9
Taking antidepressants, *n*	3
Past cognitive behavioral therapy, *n*	2

Participants’ scores for psychotic like experiences and functioning suggested that they had higher levels of symptoms and were functioning poorer when compared with larger samples of young people meeting UHR criteria (e.g., Morrison et al., 2011). In line with this, only one participant was *not* in the NEET category whilst 9/10 participants were not in education employment or training. The sample also demonstrated above cut off levels for anxiety (within the severe range) and depression (within the moderate range). Scores on the MCQ were considerably higher than equivalent populations (e.g., Bright et al., 2018), indicative of a sample who had higher levels of psychopathology than seen in previous trials of other interventions e.g., CBT.

Of the eight participants who completed the 12-week assessments, three were still at risk of psychosis, four no longer met ARMS criteria, and one had transitioned to a first psychotic episode, although declined to be referred for further treatment. At the 6 months follow-up (*n* = 6), two clients were at-risk of developing psychosis and four no longer met ARMS criteria. Summary statistics for primary and secondary outcomes (mean, *SD*) and effect size analyses (Cohen’s *d*) are presented in Table 2. In summary, at 12 weeks participants had significantly lower scores on four out of six CAARMS subscales: non-bizarre ideas (*p* = 0.018), perceptual abnormalities (*p* = 0.026), disorganized speech (*p* = 0.043), suicidal behavior (*p* = 0.042). HADS scores were significantly lower (anxiety: *p* = 0.017, depression *p* = 0.046), as were CAS-1 scores (worry: *p* = 0.018, threat monitoring: *p* = 0.028). IVI scores were significantly lower (*p* = 0.027) and GAF scores significantly higher (*p* = 0.035).

**TABLE 2:** Summary statistics and outcome data for key variables.

**Variable**	**Baseline**	**End of therapy**	**Follow-up**	**Pre to post treatment^∗^**			**Pre to follow up**		
					
	**Mean (*sd*)**	**Mean (*sd*)**	**Mean (*sd*)**	***z***	**p**	**d**	**z**	**p**	**d**
CAARMS									
Unusual thought content	12.6 (6.6)	10.4 (10.4)	4.0 (4.6)	−0.94	0.345	0.33	−1.83	0.068	1.30
Non-bizarre ideas	15.4 (5.9)	8.9 (3.3)	5.7 (3.9)	**−2.38**	**0.018**	**1.10**	**−2.21**	**0.027**	**1.64**
Perceptual abnormalities	10.7 (4.8)	7.3 (5.7)	7.8 (7.2)	**−2.23**	**0.026**	**0.71**	−0.95	0.340	0.60
Disorganized speech	9.0 (7.9)	4.3 (7.1)	5.0 (5.0)	**−2.02**	**0.043**	**0.59**	−0.95	0.340	0.52
Aggressive behavior	11.1 (7.7)	5.3 (3.2)	9.2 (5.8)	−1.82	0.068	0.75	−0.11	0.916	0.25
Suicidal behavior	7.3 (7.5)	2.0 (3.0)	5.0 (7.8)	**−2.03**	**0.042**	**0.71**	−0.73	0.465	0.30
GAF	45.3 (7.6)	58.6 (15.3)	57.8 (12.4)	**−2.10**	**0.035**	**1.75**	−1.78	0.075	1.64
HADS									
Anxiety	16.2 (3.5)	10.9 (2.1)	10.2 (3.2)	**−2.39**	**0.017**	**1.51**	**−2.02**	**0.043**	**1.71**
Depression	11.8 (6.0)	8.4 (2.9)	8.3 (4.5)	**−1.99**	**0.046**	**0.57**	−1.75	0.080	0.58
CAS-1									
Worry	7.0 (1.7)	3.3 (1.4)	4.3 (2.0)	**−2.38**	**0.018**	**2.18**	−1.84	0.066	1.59
Threat monitoring	6.6 (1.8)	2.9 (1.7)	3.5 (2.2)	**−2.20**	**0.028**	**2.06**	−1.80	0.072	1.72
MCQ-30									
Total score	90.1(12.6)	66.8 (20.3)	60.2 (12.2)	**−2.34**	**0.018**	**1.85**	**−2.20**	**0.028**	**2.37**
Positive beliefs about worry	14.3 (5.1)	14.6 (4.1)	11.0 (2.8)	−1.37	0.172	0.06	−1.83	0.068	0.65
Negative beliefs about uncontrollability & danger	20.8 (3.6)	12.7 (4.5)	12.2 (2.5)	**−2.38**	**0.018**	**2.25**	**−2.23**	**0.026**	**2.39**
Cognitive confidence	18.3 (4.8)	14.6 (5.2)	13.5 (4.3)	**−2.37**	**0.018**	**0.77**	**−2.23**	**0.026**	**1.00**
Negative beliefs about the need to control thoughts	18.2 (4.6)	13.5 (6.4)	9.5 (2.2)	**−2.37**	**0.018**	**1.02**	**−2.21**	**0.027**	**1.89**
Cognitive self-consciousness	18.5 (3.6)	13.7 (4.8)	14.0 (3.2)	−1.36	0.173	1.33	−0.96	0.336	1.25
Beliefs About Paranoia Scale	48.4 (7.4)	41.7 (13.1)	37.5 (7.9)	−1.36	0.173	0.91	−1.58	0.114	1.47
IVI	55.7 (16.6)	43.2 (16.6)	36.3 (12.0)	**−2.21**	**0.027**	**0.75**	**−2.21**	**0.027**	**1.17**

*^∗^One participant failed to complete questionnaire measures. Key: CAARMS, Comprehensive Assessment of At-Risk Mental States; GAF, Global Assessment of Functioning; HADS, Hospital Anxiety and Depression Scale; CAS-1, Cognitive Attentional Syndrome; MCQ, Metacognitive Beliefs Questionnaire; IVI, Interpretation of Voices Inventory. Bold values signify statistically significant values.*

At 6 months, IVI scores remained significantly lower (*p* = 0.027) whilst only two subscales described above remained significantly lower: CAARMS non-bizarre ideas (*p* = 0.027) and HADS anxiety (*p* = 0.043). There were no significant differences at 12 weeks or 6 months on CAARMS unusual thought content (*p* = 0.345 and *p* = 0.068, respectively) or aggressive behavior subscales (*p* = 0.068 and *p* = 0.916, respectively). There were also no significant differences in BAPS score at 12 weeks (*p* = 0.173) or 6 months (*p* = 0.114).

In line with the mechanism of change, MCQ-30 total scores were significantly reduced at 12 weeks (*p* = 0.018) and 6 months (*p* = 0.028), as were three out of five subscales: negative beliefs about uncontrollability and danger (12 weeks: *p* = 0.018, 6 months: *p* = 0.026), cognitive confidence (12 weeks: *p* = 0.018, 6 months: *p* = 0.026), and negative beliefs about need to control thoughts (12 weeks: *p* = 0.018, 6 months: *p* = 0.027). There were no differences at either time-point on positive beliefs about worry (12 weeks: *p* = 0.172, 6 months: *p* = 0.068) or cognitive self-consciousness (12 weeks: *p* = 0.173, 6 months: *p* = 0.114).

**FIGURE 2:**
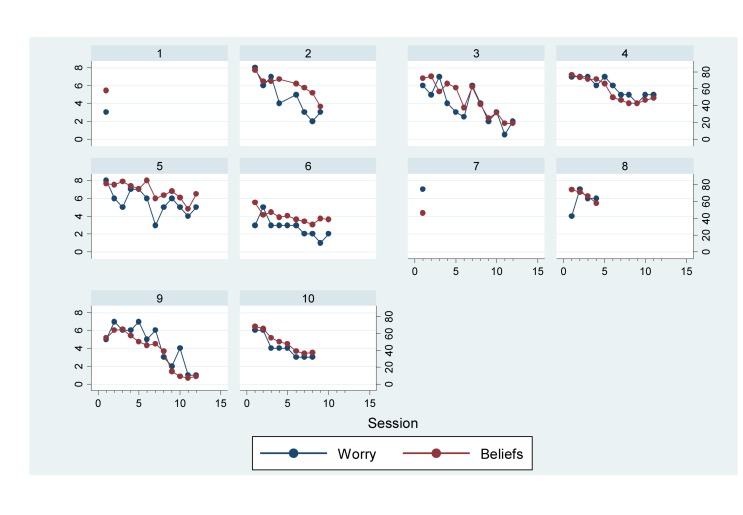
Line graph of worry and metacognitive belief scores across sessions (graphs by participant).

Successful completion of sessional measures was high (98.75%). As can be seen in Figure 2, CAS-I worry scores generally declined over the course of therapy, which coincided with reductions in CAS-I meta-cognitive belief scores. Regression, with clustering at the participant level, suggested that the strength of metacognitive beliefs significantly predicted levels of worry across the sessional measures (β = 0.73, *SE:* 0.07, *p* < 0.001, *CI:* 0.58–0.89).

## Discussion

Our results suggest that MCT is an acceptable treatment for young people with an At Risk Mental State, evidenced by high rates of trial uptake and therapy adherence (80% treatment adherence and an overall average of eight sessions). We observed clinically significant reductions in psychotic like experiences at the post-treatment assessment (CAARMS subscales: Non-Bizarre ideas, Perceptual abnormalities, disorganized speech), retained on one subscale (Non-Bizarre ideas) at the 6-month follow-up point. We also found an important reduction in participants meeting ARMS criteria at the post-treatment assessment (four out of eight) which was retained at the 6-month follow-up. Only one participant made transition to first-episode psychosis across the follow-up period. Trial results also showed statistically significant improvements in anxiety and depression (Hospital Anxiety and Depression Scale) and functioning (Global Assessment of Functioning) at the post-treatment assessment. The significant change in anxiety, but no other secondary outcomes, was retained at the 6-month follow-up assessment. Our effect sizes suggest that the magnitude of the differences found are considered medium to large, although this must be interpreted with caution as given the trial design and small sample the effect sizes are likely to be inflated.

Measures related to beliefs and processes targeted within MCT were assessed over time and during treatment. Results demonstrated clinically significant change on metacognitive beliefs (MCQ-30 total score, beliefs about uncontrollability, need for control and danger and cognitive confidence) at both follow-up assessment points. This was also the case for people’s self-rated beliefs about voices, on a measure (IVI) informed by a metacognitive model. We also observed statistically significant change on processes described within the CAS (worry and threat monitoring) at the post-treatment assessment, although this was not retained at 6 months. As with the previous outcomes, effect sizes on these measures demonstrated medium to large effects across assessments, although the effect sizes need to be interpreted with caution as previously described. Weekly measurement of worry and threat monitoring (recorded via CAS-1 subscales) showed that both reduced, at similar rates, throughout the course of treatment for those who were retained. These results suggest that MCT based upon a specific metacognitive model is capable of changing metacognitive beliefs and processes; which are hypothesized mechanisms within the model and therefore important treatment targets.

These positive results suggest that MCT appears to show potential in reducing psychotic like experiences, anxiety and depression, and increasing functioning for young people at UHR of psychosis. MCT is a relatively brief treatment compared with CBT, and therefore could be a useful treatment within of the contexts of resource restrictions and the importance of timely interventions for young people. It is not possible to conclude from such a small sample about who may benefit the most from CBT versus MCT, or indeed who could be recommended for either treatment. However, if a definitive trial were to replicate our findings this could provide rationale for service users to be offered choice from a range of evidence-based treatments.

We did, however, observe no statistically significant differences in subscales related to psychotic like experiences (CAARMS unusual thought content), positive beliefs about worry, cognitive self-consciousness and beliefs about paranoia. The associated effect sizes for cognitive self-consciousness and beliefs about paranoia were medium to large, small to large on the CAARMS unusual thought content subscale, so it is possible that these findings are related to a lack of statistical power. There appeared to be little effect on positive metacognitive beliefs at both assessment points. This may reflect the relative shortness of attended therapy sessions (average of 8 sessions), and given that modification of positive metacognitive beliefs takes place in the later phase of therapy, it may not have been addressed adequately within the treatment window. It may be that, for this population, modifications should be explored in the length of treatment window offered in order to address this given the documented importance of assertive outreach principles in this area (Morrison, 2017). It is also possible that the relative inexperience of the therapists meant that the efficiency normally derived from MCT was not achieved in this study. As adherence and competency measures were not taken during the trial it’s not possible to know if this is the case.

Acceptability of the MCT was high, with eight of ten participants adhering to treatment (operationalized as attendance to least four sessions). The remaining two participants withdrew early (after a single session) due to changes in life circumstances making attendance at therapy sessions difficult (physical health complications and moving out of area). We observed acceptable retention at the post-treatment assessment (80% completion) but higher rates of attrition at the 6-month follow-up (40%). The reasons for two participants not attending the follow-up assessment (described above) were unrelated to the trial procedures. However, two additional participants declined to take part in the 6-month follow-up assessment and it was not possible to explore their reasons for declining. Therefore, we cannot comment on any possible acceptability issues related to trial procedures for either of these participants. It will be important for future trials to explore the acceptability of trial procedures via qualitative interviews or feedback processes.

As would be expected in an exploratory trial of this kind there are a number of important methodological limitations that require consideration. The small sample size both reduces the statistical power and the generalizability of the findings, in part because our sample size did not meet statistical requirements (e.g., normality) required for hypothesis testing (e.g., Shader, 2015). Small sample sizes also have a higher risk of providing imprecise estimates and therefore we must be highly cautious when interpreting the meaning of these results to the wider population. However, MCT has been found to produce large effects in other groups (e.g., GAD treatment), and therefore is consistent with previous findings. Given the limitations of our study, we are limited in being able to compare our findings to those of larger studies of psychological interventions for ARMS populations, and more data on MCT for ARMS populations is required for verification. However, in line with the aims of pilot studies, we were able to examine the feasibility of processes and procedures (i.e., recruitment, retention, implementation of MCT) in preparation for a larger RCT (Leon et al., 2011).

The trial design did not allow for comparison with a control group, and the assessors were not blind to the presence of the treatment; therefore rater bias and possible non-specific treatment effects have not been guarded against Additionally, we did not complete formal ratings of treatment adherence or therapist competency. This limits any analysis of fidelity to the treatment protocol, although therapists did audio-tape sessions (where consent allowed) and receive supervision by the first author following the treatment manual previously described. A further limitation is that we did not obtain any qualitative feedback from participants on their views of the treatment, or any satisfaction scores. Nonetheless, we found significant effects on a number of outcome measures and potential mechanisms implicated within the MCT model, some of which were derived from self-rated questionnaires which showed statistically significant change with associated medium to large effects.

MCT for individuals meeting ARMS criteria warrants further attention. In the future, it will be important to conduct another pilot trial to further test of the acceptability and feasibility of offering MCT compared with treatment-as-usual under randomized conditions and with a longer follow-up period. The CAARMS assessment seems to be an appropriate outcome measure and would allow for comparison with other trials of ARMS interventions. Further qualitative work is required to explore participants’ unique experiences of the trial, and obtain their views on the appropriateness of the CAARMS as a primary outcome measure in a future definitive trial.

## Ethics Statement

This study was carried out in accordance with the recommendations of A National Research Ethics Committee, North West Greater Manchester West Ethics Committee (Ref. 13/NW/0238) with written informed consent from all subjects. All subjects gave written informed consent in accordance with the Declaration of Helsinki. The protocol was approved by the North West Greater Manchester West Ethics Committee.

## Author Contributions

SP conceived and designed the study. SP, PM, LM, and JP-C provided the therapy. SP and SB supervised the study. SP, PM, LM, and JP-C performed the data collection. JP-C analyzed the data. SP, JP-C, and LM interpreted the data. SP, LM, JP-C, and SB drafted the manuscript. All authors revised and finalized the manuscript.

## Conflict of Interest

The authors declare that the research was conducted in the absence of any commercial or financial relationships that could be construed as a potential conflict of interest.
